# MeCP2 mutations: progress towards understanding and treating Rett syndrome

**DOI:** 10.1186/s13073-017-0411-7

**Published:** 2017-02-17

**Authors:** Ruth R. Shah, Adrian P. Bird

**Affiliations:** Wellcome Trust Centre for Cell Biology, University of Edinburgh, Max Born Crescent, Edinburgh, EH16 5DS UK

## Abstract

Rett syndrome is a profound neurological disorder caused by mutations in the *MECP2* gene, but preclinical research has indicated that it is potentially treatable. Progress towards this goal depends on the development of increasingly relevant model systems and on our improving knowledge of MeCP2 function in the brain.

## Rett syndrome genetics

It is now 50 years since Andreas Rett reported his observations on 22 girls with similar clinical features, subsequently known as Rett syndrome (RTT). Classic RTT is defined by a regression phase and subsequent stabilization of diagnostic criteria, which include partial or complete loss of spoken language, dyspraxic gait and stereotypic hand movements such as ‘hand mouthing’ [1]. With very few familial cases available, it took a further 33 years before mutations affecting a protein called methyl-CpG-binding protein 2 (MeCP2) were shown to be the almost exclusive cause of this neurological disorder. The *MECP2* gene is located on the X chromosome, and RTT is classified as an X-linked dominant disorder. Thus, as expected, males are more severely affected and rarely survive infancy. Rett syndrome therefore overwhelmingly affects females, who, owing to X chromosome inactivation, have a mixture of cells that express either the wild-type or mutant version of MeCP2. This cellular mosaicism defines RTT and highlights the importance of MeCP2 for proper neuronal function. In the light of new insights into the function of MeCP2 and the novel gene therapy technologies currently being developed, here we discuss recent progress in understanding the molecular pathogenesis of this complex disease and the search for a therapy.

## Model systems for studying Rett syndrome

**Fig. 1:**
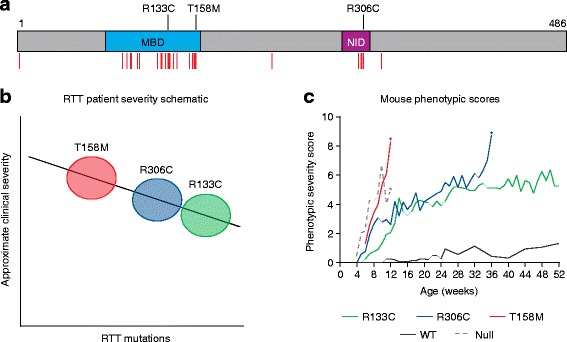
Analysis of point mutations responsible for Rett syndrome (*RTT*) in human and mouse. **a** The primary protein structure of methyl-CpG-binding protein 2 (MeCP2), which is a chromosomal protein that binds to methylated DNA, highlights two key functional domains—a methyl-CpG-binding domain (MBD) and a NCoR/SMRT co-repressor interaction domain (NID). Shown as *red vertical lines* below the schematic are the positions of all RTT-causing missense mutations (RettBASE; http://mecp2.chw.edu.au/). The positions of three particular RTT-causing missense mutations—R133C, T158M and R306C, reflecting the spectrum of clinical severity—are indicated above the schematic (modified from [6]). **b** The approximate clinical severity of patients possessing the specific missense mutations T158M (*red*), R306C (*blue*) or R133C (*green*), based on independent studies using a variety of clinical severity score systems, for example [3]. **c** Scores of phenotypic severity of mouse models containing the T158M (*red*), R306C (*blue*) and R133C (*green*) missense mutations, in comparison with those of wild-type mice (*dark gray solid line*) and *Mecp2*-null mice (*pale gray broken line*). The *asterisks* indicate where no animals of that genotype survived beyond the indicated time-point. The data are adapted from Brown et al. [2] and are reproduced with permission of Oxford University Press

Mouse models are a vital tool for studying RTT as MeCP2 deficiency closely mimics the clinical features of the human disorder, including motor defects and breathing arrhythmia. Indeed, a recent study [2] of animals expressing three *Mecp2* mutations with differing average clinical severity showed a matching severity spectrum in the mice (Fig. 1) [3]. These findings reflect the high conservation of the MeCP2 amino acid sequence between human and mouse (95% identical) and the parallel dynamics of MeCP2 expression during brain development. Thus, despite differences in the brain structure and developmental timing between human and mouse, these striking similarities suggest that the molecular consequences of MeCP2 mutation are similar between the two species. Early indications that RTT results exclusively from absence of MeCP2 in the brain have recently been reinforced by a mouse model that has normal levels of central nervous system MeCP2, but lacks this protein in the rest of the body, and shows none of the major phenotypes associated with RTT-like mice [4].

Advances in cellular technologies have allowed further development of human tissue-culture models of RTT [5, 6]. The ability to study a homogeneous population of human neurons eliminates the complexity of the brain, allows more precise genetic manipulation and simplifies the interpretation of findings [5]. It also streamlines screening of potential therapeutics and viral delivery vectors in a human neuronal setting. In addition to mice, a *Mecp2*-null rat model has been produced (Sage Labs), and there has been progress towards generating non-human primate models of RTT [7] that should be beneficial for testing once further phenotypic characterization is available.

## MeCP2—a genome-wide transcriptional repressor

Progress in understanding the molecular aetiology of RTT has been facilitated by combining data from clinical genetics and mouse models with cellular and biochemical investigations. Importantly, nearly all RTT missense mutations cluster in two discrete domains of MeCP2: the methyl-CpG-binding domain (MBD) and the NCoR/SMRT co-repressor interaction domain (NID) (Fig. 1) [6]. With a crystal structure of the MBD bound to methylated DNA available, we can understand the loss of DNA binding caused by RTT mutations in the MBD (reviewed in [6]). Likewise, most amino acids in the NID—which interact with NCoR/SMRT co-repressor complexes—when mutated cause RTT syndrome, and all NID mutations tested so far prevent the interaction with this large multi-component complex. The NCoR/SMRT complex includes the transcriptional-repression-associated histone deactylase HDAC3, thus supporting the hypothesis that MeCP2 recruits this complex to methylated sites within the genome to downregulate transcription (reviewed in [6]).

The notion that MeCP2 recruits gene silencing machinery is well supported by a variety of experimental data, but it has taken time to link this model convincingly to the effects of MeCP2 loss on transcription patterns in the brain. Recent studies, however, have uncovered striking three-way proportionalities between DNA methylation levels, MeCP2 binding-site occupancy and transcriptional inhibition [8, 9]. This progress has depended on new information regarding the DNA binding-site specificity of MeCP2 showing that the di-nucleotide methyl-CA (mCA), as well as the canonical methyl-CG (mCG), both constitute target sites. In addition, improved mapping of MeCP2 binding in vivo using chromatin immunoprecipitation has increasingly taken account of the high frequency of mCG and mCA (~1 per 100 bp) in the neuronal genome, which rendered conventional peak-finding approaches problematic. Finally, well-controlled and highly replicated RNA-sequencing analysis is required for sensitive transcriptional profiling. What has emerged from these precision studies is that MeCP2 exerts a restraining effect on transcription. This effect is global, gene-body-dependent and subtle, but correlates well with DNA methylation density, in particular affecting a large proportion of brain-specific genes that are unusually long [8, 9] (reviewed in [6]). Thus, in contrast to early expectations that MeCP2 would alter the expression of a few discrete target genes, new results portray it as a global transcriptional repressor that modulates gene expression programmes in a DNA-methylation-dependent manner. It remains unclear, however, how loss of this genome-wide modulation of gene expression patterns contributes to the observed RTT pathologies.

## Reversibility and the search for a cure

A major contribution of mouse models has been the proof-of-principle demonstration that RTT is a reversible (i.e. curable) disorder. Restoration of the gene in *Mecp2*-deficient animals with advanced Rett-like signs led to dramatic restoration of normal breathing, mobility and other features (reviewed in [6]). These results have given great impetus to the search for a cure. One approach is to seek small molecules that reverse the downstream consequences of MeCP2 deficiency, whereas an alternative is to rectify the genetic cause of the disorder through gene therapy, protein replacement or gene correction [1].

As a global regulator that tunes gene expression, and perhaps reduces transcriptional noise, it is feasible that a loss of MeCP2 function causes a transcriptional imbalance leading to a ‘sub-optimal’ brain. This underlying mechanistic heterogeneity has implications for therapeutic approaches for RTT. With many genes mildly misregulated, it might be difficult to identify a single pathway that is susceptible to small-molecule regulation that could cure RTT. More positively, it might be possible to identify drugs to target certain biochemical pathways that are crucial for brain function, and thereby ameliorate a number of aspects of RTT. Pharmacological readjustment of key pathways could be of therapeutic value, even if many other processes remain un-corrected [1]. In support of this argument, a screen for genetic suppressors of the mouse Rett-like phenotype detected multiple hits (cited in [6]), indicating that inhibition of specific pathways can indeed be beneficial. These results indicate that it is premature to rule out small-molecule-based therapeutic approaches at this stage.

Two laboratories have shown that delivery of the *Mecp2* gene using an adeno-associated virus (AAV) vector is efficient enough to rescue some of the phenotypes of *Mecp2*-null male and heterozygous female mice (reviewed in [1]). Initial concerns about inducing MeCP2 overexpression syndrome, especially in female mice that express a wild-type copy of the gene in approximately half of their cells, have so far been ameliorated by the use of endogenous promoters and systemic injection routes. There is a long way to go, but initial results suggest that gene therapy could be of clinical utility. The most desirable therapeutic scenario of all would be to correct the point mutation itself using CRISPR–Cas9 or related techniques. In vivo gene disruption of the *Mecp2* allele in wild-type mice using AAV vectors to deliver Cas9 and single guide RNA (sgRNA) has been successful, achieving a knockout of MeCP2 in approximately 70% of cells in the hippocampus [10]. This and other findings raise the possibility that in vivo correction of *MECP2* mutations will one day be feasible. To achieve targeting specificity, the RTT mutation itself (or single-nucleotide polymorphisms between the two alleles) could be used to distinguish mutant and wild-type copies of *MECP2*, and thus encourage targeting of only the mutant *MECP2* allele. Technical problems remain—most notably the restricted payload of AAV vectors requires the simultaneous use of multiple viruses for delivery of the many, large gene-editing components, which would reduce the efficiency of such an approach. Despite these current limitations, gene editing remains a promising therapeutic option.

## Concluding remarks

Pre-clinical research strongly suggests that RTT could be one of the first curable neurological disorders. The application of in-depth, genome-wide approaches and increasingly sophisticated model systems are painting a clearer picture of the functional role of MeCP2 in neurons, and this new information promises to guide future therapeutic strategies. At the same time, multiple therapies are currently being developed—for example gene therapy, reactivation of the inactive X chromosome and modulation of neurotransmitter signalling pathways. As to which of these diverse approaches could be of therapeutic value, only time will tell.

## Abbreviations

AAV: Adeno-associated virus
Cas9: CRISPR-associated protein 9
CRISPR: Clustered regularly interspaced short palindromic repeat
mCA: Methyl-CA
mCG: Methyl-CG
MDB: Methylated-DNA binding domain
MeCP2: Methyl-CpG-binding protein 2
NID: NCoR/SMRT co-repressor interaction domain
RTT: Rett syndrome
sgRNA: Short guide RNA
SNP: Single-nucleotide polymorphism
